# Lactic Acid and Salt Separation Using Membrane Technology

**DOI:** 10.3390/membranes11020107

**Published:** 2021-02-03

**Authors:** Sahar Talebi, Michael Garthe, Florian Roghmans, George Q. Chen, Sandra E. Kentish

**Affiliations:** 1 The ARC Dairy Innovation Hub, Department of Chemical Engineering, University of Melbourne, Parkville, VIC 3010, Australia; stalebi@student.unimelb.edu.au (S.T.); gechen@unimelb.edu.au (G.Q.C.); 2 Chemical Process Engineering, RWTH University, Forckenbeckstraße 51, 52074 Aachen, Germany; michael.garthe@rwth-aachen.de (M.G.); Florian.Roghmans@rwth-aachen.de (F.R.)

**Keywords:** dairy, lactic acid, reverse osmosis, electrodialysis

## Abstract

Acid whey is a by-product of cheese and yoghurt manufacture. The protein and lactose within acid whey can be recovered using nanofiltration and electrodialysis, but this leaves a waste stream that is a mixture of salts and lactic acid. To further add value to the acid whey treatment process, the possibility of recovering this lactic acid was investigated using either low energy reverse osmosis membranes or an electrodialysis process. Partial separation between lactic acid and potassium chloride was achieved at low applied pressures and feed pH in the reverse osmosis process, as a greater permeation of potassium chloride was observed under these conditions. Furthermore, lactic acid retention was enhanced by operating at lower temperature. Partial separation between lactic acid and potassium chloride was also achieved in the electrodialysis process. However, the observed losses in lactic acid increased with the addition of sodium chloride to the feed solution. This indicates that the separation becomes more challenging as the complexity of the feed solution increases. Neither process was able to achieve sufficient separation to avoid the use of further purification processes.

## 1. Introduction

Lactic acid is a valuable organic acid used in the food, pharmaceutical, leather, textile, and chemical industries. It is commercially produced either by the hydrolysis of lactonitrile or microbial fermentation. The chemical synthesis pathway is costly as it involves several distillation steps [1]. On the other hand, the microbial fermentation pathway is economically attractive due to the possibility of using low-cost raw materials [2]. However, the process requires the addition of nutrients, sugars and microorganisms to the reaction media.

Acid whey, a by-product of cream cheese and yogurt manufacturing processes, is known to contain high concentrations of lactic acid (5.5–6 g/L) [3,4]. The presence of the lactic acid can affect the drying of acid whey [5,6]. Therefore, many researchers have examined the use of different membrane technologies, such as nanofiltration (NF), dia-nanofiltration (Dia-NF) and electrodialysis (ED), to remove 30–80% of the lactic acid originally present [4,7,8]. These processes leave a waste stream that represents an unutilized source of lactic acid.

The recovery of lactic acid from acid whey has not been studied in the literature. The closest concept is the recovery of lactic acid from fermentation broths where the solution is made of sodium lactate, glucose, yeast extract, and low concentrations of other charged ions [9,10,11,12,13,14]. However, the waste streams generated from the acid whey treatment process are known to contain many of the minerals found originally in acid whey. This reduces the purity of the lactic acid that can be recovered, limiting its applications and complicating the purification process.

To separate lactic acid from a salt solution, several researchers have investigated the use of pressure driven membrane technology, such as NF. NF membranes are known for their ability to separate components based on both size and charge [15,16]. However, due to the high molecular cut-off of these membranes (200–1000 Da), both lactic acid and monovalent salts tend to pass through the membranes. In addition, several other factors affect the lactic acid permeation through NF, namely feed solution pH, process temperature, membrane material, the concentration of lactic acid in the feed, and mineral solubility [17]. In particular, an important factor that affects the permeability of lactic acid is the ratio of lactate ions to non-ionized (non-dissociated) lactic acid in the feed stream. This ratio depends strongly on the equilibrium of lactic acid at the operating temperature of the system and can be calculated using the Henderson–Hasselbalch equation (Equation (1)).

(1) $$\mathrm{pH}=pK_{a}+\log\frac{\left[lactate\right]}{\left[lactic\;acid\right]}$$

where, *pK_a_* is the dissociation constant of lactic acid, which is 3.86 at 25 °C [18].

Although the separation of lactic acid from a salt solution using reverse osmosis (RO) membranes has not been investigated, RO membranes have been used widely for the recovery or separation of other organic acids as summarized in Table 1. Considering that lactic acid has a molecular weight (MW) of 90 g/mole, this Table suggests that it should be rejected by most RO membranes.

Electrically driven membranes, such as ED, have also been widely investigated for the purification of lactic acid from fermentation broths [9,19,20,21]. Thang et al. [22] were one of the few research groups to have investigated the recovery of lactic acid from a complex solution containing high concentrations of charged species. The authors showed that operating the ED unit at low pH assisted in retaining the lactic acid in the diluate stream in the first 80 min of the process. The authors did not report the purity of the produced lactic acid, however, their results demonstrated that sulfate and divalent cations were not fully removed from the diluate stream.

This work investigates the use of both RO membranes and ED for the separation of lactic acid from potassium chloride (KCl), as an indication of the potential for recovery from acid whey. Low energy ‘loose’ RO membranes, with properties intermediate between NF and RO were chosen, with the aim that KCl would permeate, while lactic acid was retained. In addition, the effect of the presence of other salts on the retention of lactic acid within the ED diluate was evaluated by the addition of sodium chloride (NaCl) to the feed stream.

## 2. Materials and Methods

### 2.1. Materials

Purified water (>8.6 MΩ cm; Merck Millipore KGaA, Darmstadt, Germany), lactic acid (85–90%; Thermo Fisher Scientific, Scoresby, Australia), KCl (>99.0%; Chem-supply, Gillman, Australia), and NaCl (>99.5%; Merck KGaA, Darmstadt, Germany) were used in the preparation of the feed solution. For the ED experiments, 20 g/L sodium sulfate (Na_2_SO_4_; >99%; Thermo Fisher Scientific Australia Pty., Ltd., Scoresby, Australia) was used as the electrolyte solution.

Three commercial low energy RO membranes were used, namely LE and XLE (DuPont Filmtec, Edina, MN, USA), and AK (Suez, Minnetonka, MN, USA). Both LE and XLE membranes are specified to have a salt rejection of 99% but the former is for a feed solution of 2000 ppm NaCl at an applied pressure of 10.3 bar; while the latter is for a feed solution of 500 ppm NaCl at an applied pressure of 6.9 bar. Both membranes are strongly negatively charged at neutral pH, with an isoelectric point of 4.3 [27,28]. The AK membrane is also specified to have a salt rejection of 99% for a feed solution of 500 ppm NaCl at an applied pressure of 7.9 bar. The membrane is negatively charged at neutral pH, but the isoelectric point is not provided by the manufacturer.

For the ED experiments, cation exchange membranes (Neosepta CMB) and anion exchange membranes (Neosepta AHA) were purchased from Astom Co., Ltd. (Tokyo, Japan). These membranes have a wide pH tolerance and thermal stability. Information on the membrane characteristics can be found elsewhere [4].

### 2.2. Reverse Osmosis (RO) Membrane Experiment Setup and Protocol

**Figure 1:**
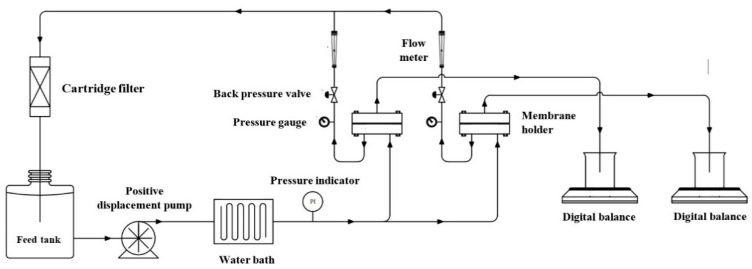
The experimental arrangement used for the reverse osmosis (RO) experiments.

The experiments for lactic acid and salt separation were carried out in a crossflow configuration in duplicate (Figure 1), using two parallel flat sheet membrane modules (CF042 Sterlitech Corporation). The membrane active area in each cell was 4.6 cm × 9.2 cm. The unit consisted of a 60 L feed tank; Hydra-Cell G10 positive displacement pump (Wanner Engineering, Minneapolis, MN, USA) with back pressure regulator (Hydra-Cell C62 series, Wanner Engineering Minneapolis, MN, USA); a guard filter (5 µm carbon filter, Puretec, Lonsdale, SA, Australia) in the retentate line to eliminate contaminants from re-entering the system; water bath to maintain the temperature; digital pressure indicator (Ashcroft, Stratford, CT, USA) to monitor the feed pressure; flow meters (Blue-White Industry, Huntington Beach, CA, USA), and analogue pressure gauges (Floyd Instruments, Tullamarine, Australia) to monitor the retentate pressure. More details are provided in Hoang et al. [29].

Before conducting the experiments, the membranes were soaked in water to allow membrane hydration. Once the membranes were placed inside the membrane cell, the membranes were compacted under 30 bar feed pressure. The experiments were performed at 25 ± 1 °C, using feed solutions with different concentration of KCl and lactic acid with or without pH adjustment. The applied pressure during the experiments ranged from 6 to 30 bars. For each experiment, 40 L of feed solution was used to minimize fluctuations in feed conditions. Samples were taken continuously from the feed tank and analyzed for lactic acid concentration, pH, and conductivity to ensure uniform feed conditions throughout the run. For each applied pressure, the system was stabilized for at least 30 min before a permeate sample was taken. A second sample was taken after a further 30 min to ensure data reproducibility. If a difference was noted between these two samples then a third measurement was taken. The permeate flux was measured at two different cross flow velocities of 1.1 cm/s and 1.3 cm/s, respectively.

### 2.3. Electrodialysis (ED) Experiments Setup and Protocol

ED refers to the process in which ions are transferred through ion exchange membranes under the application of an electric field. Three processing streams are utilized in an ED process: (1) a diluate stream from which ions are removed; (2) a concentrate stream that uptakes the ions lost by the diluate stream; and (3) an electrolyte solution that conducts the current through the electrode compartments and protects the electrodes.

**Figure 2:**
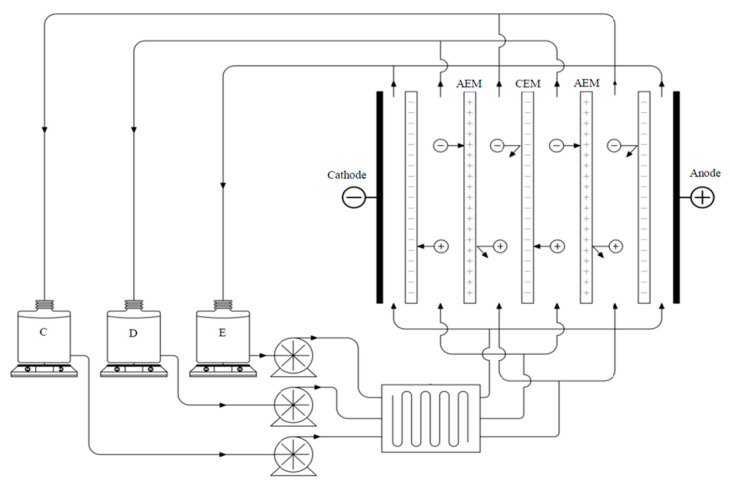
The experimental arrangement used for the electrodialysis (ED) experiments ((C = concentrate, D = diluate, E = electrolyte, CEM = cation exchange membrane, AEM = anion exchange membrane).

The electrodialysis experiments were conducted using an FTED-40 module manufactured by FuMA-Tech GmbH (Bietigheim-Bissingen, Germany). The ED unit consisted of two titanium-iridium plasma coated stainless steel electrodes. The module was arranged with two CMB membranes separated by two AHA membranes and alternating diluate and concentrate spacers. A third CMB membrane was placed in front of the electrodes to prevent the migration of anions into the electrode compartment. The effective area per membrane was 36 cm^2^. The potential difference was generated using a direct current (DC) power supply (Agilent DC Modular Power System N6764A) with an output voltage range between 0–60 V and an output current range between 0–20 A. Three peristaltic pumps (Masterflex L/S digital drive 600 RPM with Masterflex L/S high performance pump head) were used to circulate the electrolyte, concentrate and diluate solution through the ED unit (Figure 2). The diluate and concentrate flowrates were maintained at 500 mL/min, while the electrolyte flowrate was kept at 1000 mL/min as recommended by the manufacturer to minimize concentration polarization effects. The solutions were stirred continuously throughout the experiments. A water bath was used to maintain the temperature of the solutions at 25 ± 1 °C. One litre of solution was used for the diluate and concentrate streams respectively. The initial composition of the concentrate solution was identical to the diluate solution to minimize mass transfer by diffusion between the two streams. Experiments were performed at room temperature under a constant voltage of 2.4 V. The ED process is usually terminated at 70% demineralization of the diluate stream to avoid high energy costs [5], therefore, the results were evaluated at 70% removal of conductivity (70% demineralzation rate (DR)).

### 2.4. Analytical Methods

The permeate and feed samples from the RO membrane experiments and samples from the ED diluate tank (collected every 10–15 min) were analyzed using inductively coupled plasma optical emission spectroscopy (ICP-OES 720ES, Varian, Palo Alto, CA, USA) to measure the concentration of sodium (Na) and potassium (K). High-performance liquid chromatography (HPLC, Shimadzu, Kyoto, Japan) was used to measure lactic acid concentrations in the collected samples. All samples were filtered and diluted according to the equipment detection limits. Information on the operating parameters of the ICP-OES and HPLC can be found elsewhere [4].

#### 2.4.1. RO Membrane Experiments

The contact angle for the RO membranes were measured using First Ten Angstroms 200 (FTA200, Portsmouth, VA, USA). An image was captured using a digital recorder (Sanyo Electric VCB-3512) and analyzed using instrument software. The water flux through the RO system was measured experimentally as a function of pressure by recording the permeate mass on a balance (Pioneer Series, Ohaus, Port Melbourne, Australia). The solute rejection (*R_j_*) refers to the percentage of compounds that do not pass the membrane and was calculated based on Equation (2).

(2) $$R_{j}\left(\%\right)=1-\frac{C_{j,P}}{C_{j,F}}\times 100$$

where, *C_j,P_* and *C_j,F_* refer to the concentration of ion *j* in the permeate and feed, respectively.

#### 2.4.2. ED Experiments

The limiting current density of the ED membranes was measured for a solution of 2.5 g/L KCl and 1 g/L of lactic acid, using a six compartment electrodialysis cell. Two working electrodes were used to apply the voltage inducing electric current, while two reference electrodes recorded the voltage drop across the membrane, using Haber–Luggin capillaries to bring these close to the membrane surface. Further details of this technique are provided in Roghmans et al. and Luo et al. [30,31].

The pH and conductivity of the diluate stream was measured continuously during the ED process using a pH and conductivity meter (Mettler-Toledo, Greifensee, Switzerland). The percentage change in conductivity of the diluate tank (also known as the demineralization rate (DR)) was calculated according to Equation (3) [32].

(3) $$Change\;in\;conductivity\;\left(\%\right)=\frac{x_{initial}-x_{final}}{x_{initial}}\times 100=\left(1-\frac{x_{final}}{x_{initial}}\right)\times 100$$

where x is the conductivity of the diluate solution (µS/cm).

All error margins are based on one standard deviation either side of the mean.

## 3. Results

### 3.1. Reverse Osmosis for Lactic Acid and Salt Separation

#### 3.1.1. Membrane and Process Characteristics

**Figure 3:**
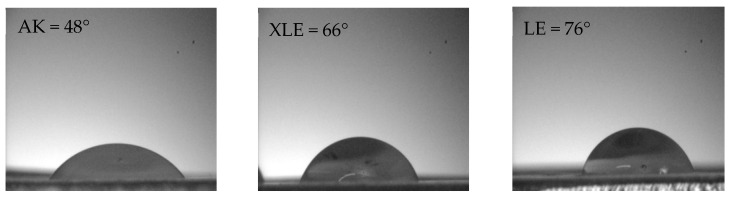
Contact angle of the loose RO membranes used in this study.

All three RO membranes used in this study were hydrophilic, with contact angles lying within the range of 48°–76° (Figure 3). The lowest contact angle was measured for the AK membrane indicating that these membranes are the most hydrophilic.

**Figure 4:**
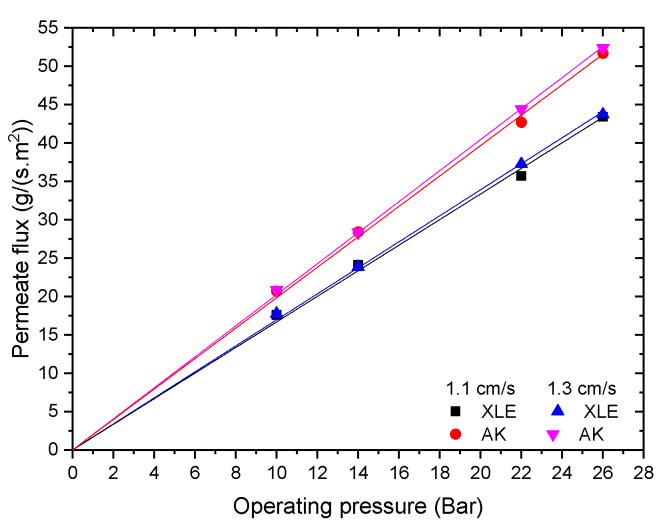
Permeate flux at two different cross flow velocities for a feed solution of 0.9 ± 0.1 g/L of lactic acid with no added KCl.

The permeate flux was measured at different applied pressures for a solution of 0.9 ± 0.1 g/L of lactic acid with no added KCl (Figure 4). No significant difference was observed for the permeate flux obtained at two different crossflow velocities. It was noted that the permeance of the XLE membrane was lower than of the AK membrane (1.6 versus 2.0 g/m^2^·s·bar). This could be partially due to the greater hydrophilicity of the AK membrane (Figure 3) thus allowing more water to pass through the membranes. However, it may also reflect a structure that is less heavily crosslinked, leading to lower rejection of different components.

#### 3.1.2. Effect of Operating Pressure on Lactic Acid Rejection

**Figure 5:**
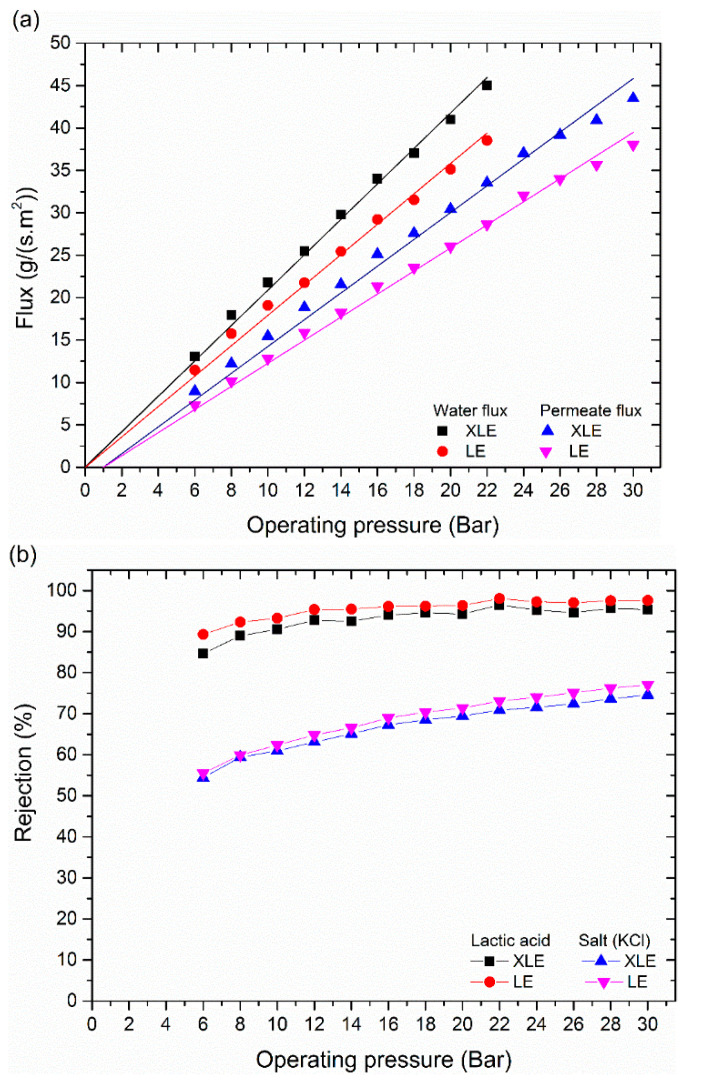
Results of experiments with Filmtec low energy membranes and a feed solution of 2.5 g of KCl and 650 ± 50 mg lactic acid per litre, pH of 3.5, conductivity of 4.8 mS/cm, and operating temperature of 25 °C: (**a**) pure water and permeate flux, and (**b**) lactic acid and KCl rejection.

The effect of operating pressure was evaluated using a feed solution made of 2.5 g/L of KCl and 650 ± 50 mg/L of lactic acid. The water and permeate flux are illustrated in Figure 5a for LE and XLE membranes. Higher flux values were observed for the XLE membrane due to its greater hydrophilicity when compared to the LE membrane (Figure 3) and possibly by differences in the free volume of the polyamide layer. Furthermore, it was observed that as the applied pressure increased, the rejection of lactic acid and KCl increased (Figure 5b) from 85 to 98% and 54 to 75%, respectively. This is a very common phenomenon and results from the fact that salt flux is independent of pressure, while water flux increases with pressure. Thus, the concentration of salt decreases in the permeate with an increase in pressure, in turn, increasing rejection.

The rejection of lactic acid was always higher than the salt present in the feed solution. The pH of the feed solution was 3.5, which means that around 44% of the lactic acid was dissociated. Further, at this pH the membrane was positively charged, given the isoelectric pH of the XLE and LE membranes is around 4.3. This means that the strong rejection of lactic acid is due to the larger molecular weight (90 g/mol) when compared to K (39.0 g/mol) and Cl (35.4 g/mol), rather than charge effects. These results are in agreement with the results reported for other organic acids in Table 1, where acids with MW equal or greater than 88 are highly retained by RO membranes, while acids with smaller molecular weight (such as boric acid and acetic acid) tend to pass through. Timmer et al. obtained 78% and 99% rejection of lactic acid from a fermentation broth using two different RO membranes, with this rejection falling to 46–67% when NF membranes were used [13]. Moreover, lactic acid has a radius of 3.13Å [33] and contains only two OH^−^ groups in its structure. This reduces the number of hydrogen bonds that can be formed with the water permeating through the membranes in comparison with, for example, boric acid which has three OH^−^ groups. The high rejection of lactic acid provides an opportunity to separate the lactic acid from the salt. Since both the LE and XLE membranes behaved similarly, the remaining experiments used XLE membranes only.

#### 3.1.3. Effect of Potassium Chloride on Lactic Acid Rejection

**Figure 6:**
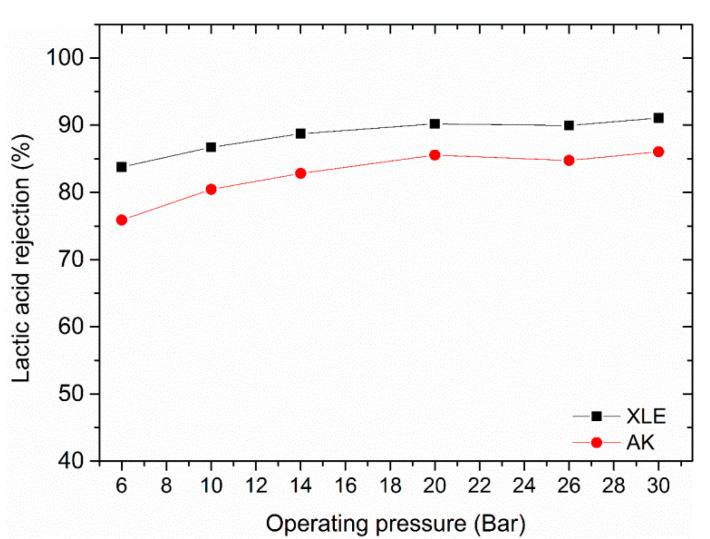
Effect of the absence of KCl on lactic acid rejection. Feed solution 0.9 ± 0.1 g/L of lactic acid, pH of 2.85 and operating temperature of 25 °C.

The high ionic strength induced by the presence of KCl could be another factor contributing to the high rejection of lactic acid. As a result, a feed solution made of 0.9 ± 0.1 g/L of lactic acid with no added KCl was investigated. It can be observed that the absence of KCl did not significantly alter the high rejection of lactic acid, as shown in Figure 6.

#### 3.1.4. Effect of Operating Temperature on Lactic Acid Rejection

**Figure 7:**
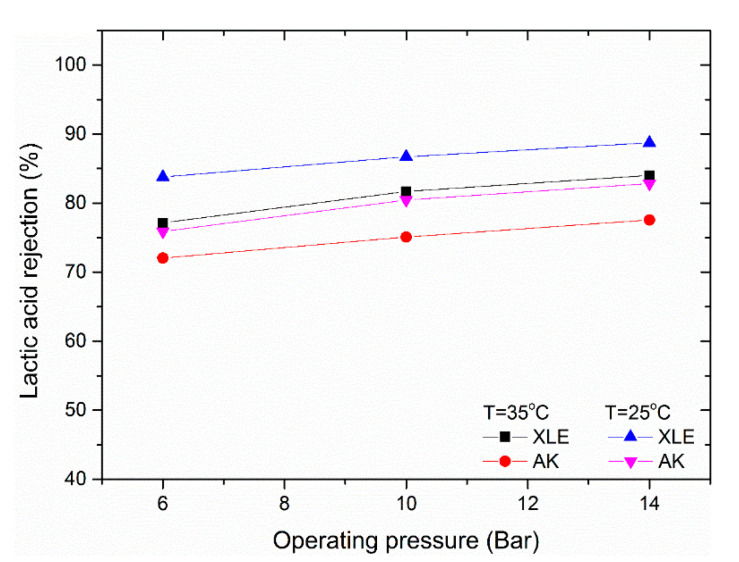
Effect of operating temperature on lactic acid rejection. Feed solution 0.9 ± 0.1 g/L of lactic acid and pH of 2.85.

To study the effect of system temperature on lactic acid rejection, experiments were conducted at 25 °C and 35 °C using a feed solution of 0.9 ± 0.1 g/L lactic acid with no added KCl. The increase in temperature from 25 °C to 35 °C decreases the *pK*_a_ value for lactic acid from 3.86 to 3.76, based on the formula provided by Perrin [34], but this only shifts the dissociation of lactic acid from 9% to 12% (Equation (1)). However, the free volume within the polymer increases, leading to greater permeability of all species. It is this second effect that most probably results in reduction in lactic acid rejection observed in Figure 7.

#### 3.1.5. Effect of Feed pH on Lactic Acid Rejection

**Figure 8:**
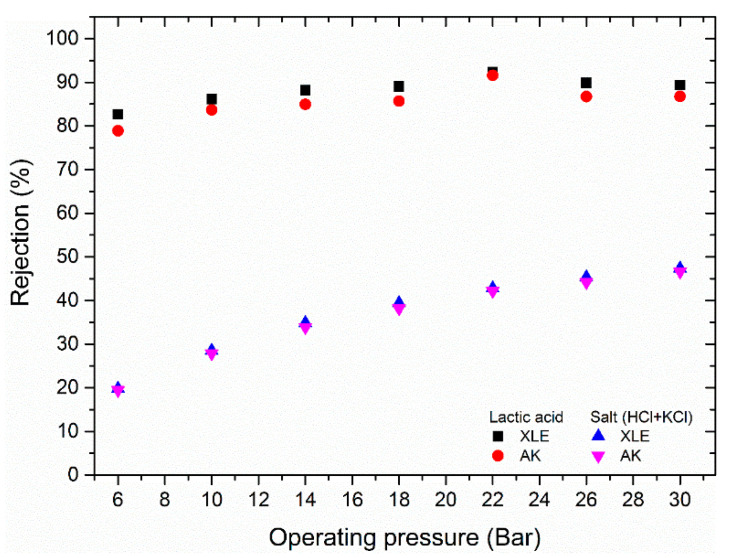
Lactic acid and salt rejection at a reduced feed solution pH of 2.3 as a function of operating pressure (operating temperature of 25 °C, conductivity of 14 mS/cm and estimated composition of 1 g/L (11 mM) of lactate, 38 mM of Cl^−^, 33 mM of K^+^, and 5 mM of H^+^).

The pH of the feed solution was decreased to 2.3 using 5 M HCl solution, increasing the feed conductivity from 4.8 to 14 mS/cm. The rejection of lactic acid did not reduce significantly with the change in feed solution pH (Figure 8) consistent with the argument that the high rejection of lactic acid is not due to charge effects, but rather size. Other workers have shown that when NF membranes of greater pore size are used, there is a significant reduction in rejection as the pH is lowered due to the dissociation of the acid [14,17].

Conversely, as the acidity of the feed solution increased, the membranes will become more positively charged. This leads to greater transmission of chloride ions and in turn, a lower rejection of both HCl + KCl. Thus, at 6 bar transmembrane pressure, 80% of the salt (HCl + KCl) permeates, whereas 80% of the lactic acid is retained. With an 80% stage cut, the retentate composition would change to 4.2 g/L lactic acid and 0.5 g/L salt (HCl + KCl); after a second stage, this would be 17.6 g/L lactic acid and 0.9 g/L salt (HCl + KCl). However, there would be a 30% loss of lactic acid into the permeate across the two stages.

### 3.2. Electrodialysis for Lactic Acid and Salt Separation

#### 3.2.1. Limiting Current Density Determination

**Figure 9:**
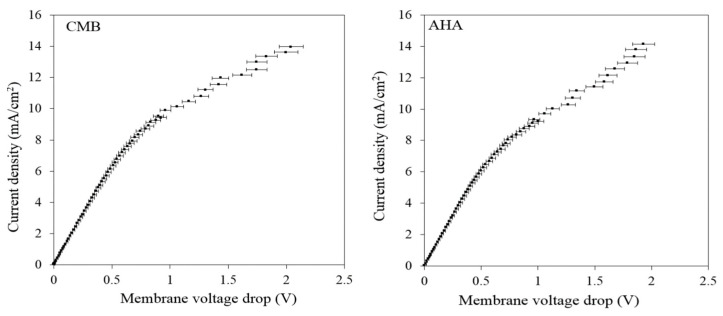
The current-voltage curve for a solution of 2.5 g/L KCl and 1 g/L lactic acid.

The limiting current density for the electrodialysis experiments was determined using a feed solution made of 2.5 g/L KCl and 1 g/L of lactic acid. The three different regions, i.e., ohmic, plateau, and overlimiting current region, are not observed clearly for the selected feed solution, as the Ohmic region merges with the limiting current region (Figure 9). This is mainly due to the low concentration or conductivity of the feed solution, thus resulting in low limiting current density. Due to the limitation with the power supply higher voltages could not be applied and thus the overlimiting region was not detected. Nevertheless, a value of 6 and 7 mA/cm^2^ was calculated for the limiting current density of the AHA and CMB membranes, respectively, from the intersection of the linearized ohmic and plateau regions of the current-voltage curve (see Roghmans et al. [30]). For the lab-scale ED unit used, the membrane area was 36 cm^2^ per membrane pair. Therefore, the limiting current for the system would be in the range of 216–252 mA. Thus, all experiments were conducted at a constant voltage of 2.4 V where the initial current was always below 216 mA and this current declined further as the solution conductivity fell.

#### 3.2.2. Effect of Lactic Acid Concentration on the Retention of Lactic Acid

**Figure 10:**
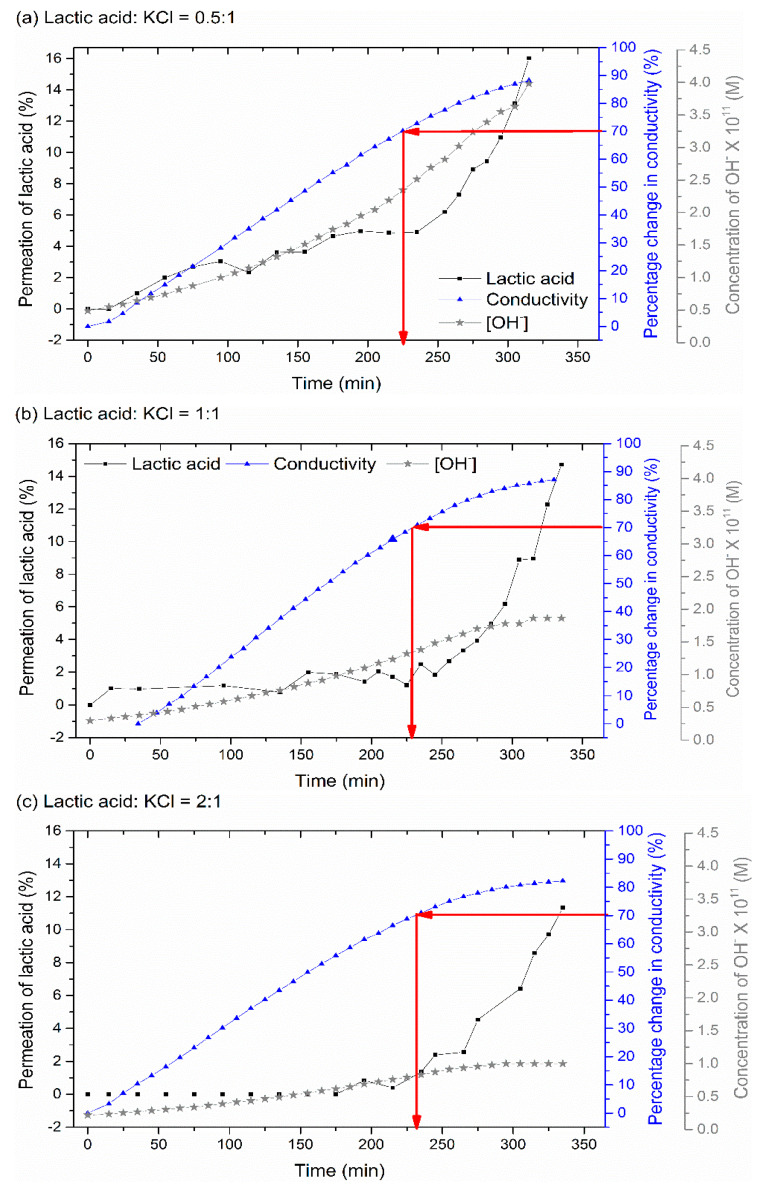
The percentage removal of lactic acid (⯀) and salt (as given by the conductivity, (▲)) from the diluate with time for three different feed compositions (**a**) Lactic acid:KCl = 0.5:1, (**b**) Lactic acid:KCl = 1:1 and (**c**) ) Lactic acid:KCl = 2:1, treated at a constant voltage of 2.4 V. The red arrows indicate the point corresponding to 70% demineralization, as measured by the diluate conductivity. Also shown is the hydroxide (OH^−^) concentration in the diluate over this period (★).

Three feed solutions with different ratios of lactic acid and KCl were examined. In all experiments, the concentration of KCl was kept constant at 1.9 g/L while the concentration of lactic acid was changed. Cl^−^ ions act as the main competitor to the lactate ion in carrying the electrical charge. As these ions are smaller and more mobile, the rate of removal (as indicated by the solution conductivity) is significantly higher than that of the lactate initially (Figure 10). However, once most of the Cl^−^ ions are removed, this driving force becomes available for the removal of lactate ions.

All three feed solutions reached 70% removal in conductivity at 225 min (Figure 10), consistent with the fact that they all contained the same concentration of KCl. The lactic acid to $K^{+}$ molar ratio improved around fourfold for all starting feed concentrations (Table 2), suggesting the approach may again be viable for separating these salts from lactic acid.

The experiment with the highest or equal concentration of lactic acid to KCl in the feed solution (1:1 and 2:1), resulted in the smallest percentage loss of lactic acid into the concentrate stream at 70% demineralization (Table 2). This can be attributed to the smaller change noted in the diluate pH. This change in pH is either a result of water splitting as the solution conductivity falls; or the migration of lactate ions and protons from the diluate to the concentrate stream [4,35]. It is well known that as the feed solution becomes less acidic, the dissociation of lactic acid to lactate increases (Table 2). This means that the electrical current is not needed anymore to split the lactic acid into lactate, but solely used to transfer the lactate ion from the diluate to the concentrate tank. This argument can be supported by considering the case when there is less lactic acid present (0.5:1) The highest concentration of OH^-^ is observed in the diluate stream (Figure 10a) which in turn results in the greatest ratio between lactate to lactic acid (0.32 as per Table 2) and the greatest loss of lactic acid.

#### 3.2.3. Effect of NaCl Addition on Lactic Acid Retention

**Figure 11:**
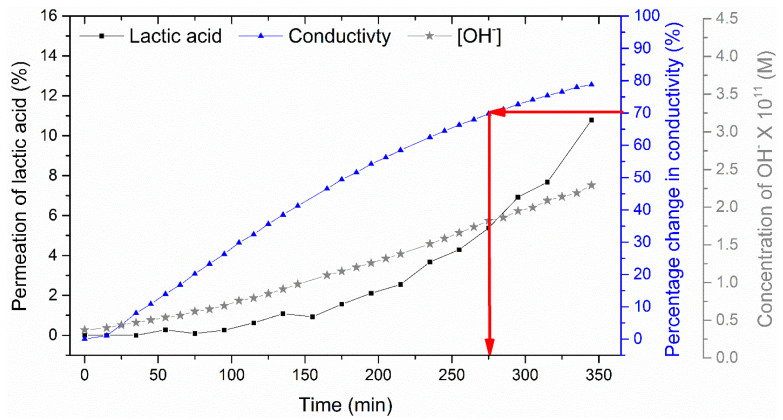
Effect of NaCl addition on lactic acid permeation at a constant voltage of 2.4 V. The red arrows indicate the point corresponding to 70% demineralisation, as measured by the diluate conductivity.

Previous work has demonstrated that ions tend to compete in electrodialysis, for example, monovalent ions have a faster migration rate when compared to divalent ions [36]. To demonstrate the effect of multiple cations on lactic acid retention, a solution was made with 1:1 mass ratio between lactic acid and total salts. The salt mixture used was 70 wt% KCl and 30 wt% NaCl, which is typical of acid whey waste. The addition of NaCl increases the time required to achieve 70% DR from 225 min (Figure 10a) to 285 min (Figure 11). The sodium ion is larger than the potassium ion and thus migrates more slowly and does not compete as readily against the lactate ion [3,36,37]. This causes both the extension in demineralization time and a greater loss of lactic acid. As shown in Figure 11**,** the addition of NaCl resulted in a 6% loss of lactic acid at 70% DR in comparison to less than 2% lost when only KCl was added to the feed solution (Figure 10a). The molar ratio of lactic acid to cations in the diluate also drops from 4.3 (Table 2) to 1.8 (Table 3) as NaCl is added. These results demonstrate that the recovery of lactic acid becomes challenging when the feed solution contains multiple ions.

## 4. Conclusions

The waste streams generated from acid whey treatment are a valuable source of lactic acid. These streams, however, are known to contain many other minerals that reduce the purity and final use of this potentially valuable ingredient. Here we have investigated the use of low energy RO and ED for the separation of the lactic acid from these minerals. Operation of the RO process at low pH and low temperature allowed for partial separation of lactic acid from KCl as the lactic acid was retained in the retentate stream with the salts permeating. The use of ED also showed some positive results as 70% KCl removal could be achieved while retaining most of the lactic acid in the feed solution. When operated under similar conditions, both ED and RO were able to produce lactic acid solutions of 90–95% purity. Thus, either the ED or RO process could be used to achieve a partial demineralization. Nevertheless, food-grade lactic acid contains minerals only at the ppm level, so the product from either approach would require further treatment using other demineralization technologies such as an ion-exchange process. The loss of lactic acid was small (of the order 1–5%) for the ED approach, whereas a two stage RO process would lead to a much greater loss of around 30%. Conversely, it is known that ED systems incur significantly greater capital cost than RO systems, due to more expensive membranes and electrodes. These competing factors would dictate the choice between RO and ED for this process.

When NaCl was added to the ED system, the permeation of lactic acid increased and the purity of the remaining lactic acid in the diluate declined. This indicates that as the complexity of the feed solution increases, the ED process becomes less effective due to the difference in the migration rate observed for different ions. While not tested in the present work, similar results might be expected in RO, as the larger sodium ion would be more strongly rejected than the potassium ion. It is anticipated that divalent ions such as calcium or magnesium, which are also commonly found in these effluent streams, could exacerbate these effects for both systems.

## Figures and Tables

**Table 1:** Separation of different organic acids using reverse osmosis membranes.

Organic Acid	Molecular Weight	Membrane Type/Salt Rejection	Feed Solution	Organic Acid Rejection	Conditions	Reference
Acetic acid	60.05	Suez (GE Osmonics) AG (Aromatic polyamide)/99.5% NaCl	7% acetic acid	25% 32%	26 Bar 40 Bar	[23]
			7% acetic acid and 15% glucose	0% 0%	26 Bar 40 Bar	
Acetic acid	60.05	Suez (GE Osmonics) CE (Cellulose acetate)/97% NaCl	7% acetic acid	−5% 0%	26 Bar 40 Bar	[23]
Boric acid	61.83	BW30LE (polyamide)	4 mg/L boron	70% 98%	12 Bar pH 4 pH 10.5	[24]
Ethyl acetate	88.10	Poly (ether/amide) membrane	366 ppm ethyl acetate	95.3%	69 Bar pH 6.0	[25]
Phenol	94.11	Poly (ether/amide) membrane	100 ppm phenol	93% ˃99%	69 Bar pH 4.9 pH 12.0	[25]
Benzoic acid	122.12	Cellulose acetate membranes/91.7% NaCl		20% 90%	10 Bar pH 3 pH 7	[26]
Citric acid	192.12	Poly (ether/amide) membrane	10,000 ppm citric acid	99.9%	69 Bar pH 2.6	[25]

**Table 2:** Experimental results at 70% demineralzation rate (DR) for a feed solution of lactic acid and KCl.

Initial Lactic Acid to KCl Mass Ratio		0.5:1	1:1	2:1
Initial concentration in the diluate (g/L)	K	0.99	1.01	1.01
	Lactic acid	1.25	2.42	4.92
Concentration at 70% DR (g/L)	K	0.21	0.24	0.23
	Lactic acid	1.19	2.38	4.84
Lactic acid to K^+^ molar ratio	Initial	0.55	1.0	2.1
	70% DR	2.5	4.3	9.1
Lactate to lactic acid ratio	Initial	0.07	0.04	0.03
	70% DR	0.32	0.18	0.11
Percentage loss in lactic acid (%)		4.8	1.7	1.6

**Table 3:** Experimental results at 70% DR for a feed solution of lactic acid, KCl and NaCl.

Initial Lactic Acid to Salt Mass Ratio		1:1
Initial concentration in the diluate (g/L)	Na	0.27
	K	0.77
	Lactic acid	2.46
Concentration at 70% DR (g/L)	Na	0.24
	K	0.15
	Lactic acid	2.31
Lactic acid to cations molar ratio	Initial	0.87
	70% DR	1.8
Lactate to lactic acid ratio	Initial	0.05
	70% DR	0.26
Percentage loss in lactic acid (%)		6

## Data Availability

The data presented in this study are available on request from the corresponding author.
